# Perioperative antibiotic prophylaxis, prostate size and transperineal prostate biopsy

**DOI:** 10.1002/bco2.70214

**Published:** 2026-05-15

**Authors:** Maxwell Louis Sandberg, Robert Smith, Randall Bissette, Madeline Sandberg, Gregory Russell, Alejandro Rodriguez, Matvey Tsivian

**Affiliations:** ^1^ Wake Forest University School of Medicine Winston Salem North Carolina USA; ^2^ Medical University of South Carolina Charleston South Carolina USA; ^3^ University of Michigan–Ann Arbor Ann Arbor Michigan USA; ^4^ Department of Biostatistics Wake Forest University School of Medicine Winston Salem North Carolina USA

**Keywords:** infection, MRI, prostate biopsy, prostate cancer, transperineal, urinary retention

## Abstract

**Introduction:**

Transperineal prostate biopsy (TPPB) is increasingly used for prostate cancer diagnosis due to low postoperative infection rates. However, the necessity of perioperative antibiotics remains debated. Although infectious complications such as urinary tract infections (UTIs) are uncommon, acute urinary retention (AUR) is a recognized complication. This study compared infection rates between patients undergoing TPPB with versus without perioperative antibiotics and evaluated rates of AUR and its association with prostate size.

**Methods:**

We conducted a multi‐institutional retrospective review of patients who underwent TPPB from 2012 to 2025. Patients were stratified by receipt of perioperative antibiotics (second‐ or third‐generation cephalosporin) versus no antibiotics. Infection was defined as fever ≥38.3°C with or without UTI symptoms or a positive urine culture (>10^5^ CFU) within 96 h of biopsy. AUR was defined as inability to void within 72 h requiring catheterization. Outcomes were compared based on antibiotic use and development of AUR.

**Results:**

A total of 904 patients were included (771 antibiotics and 133 no antibiotics). Eighteen patients (2.0%) developed infection, with no significant difference based on antibiotic usage (*p* > 0.05). Patients who developed infection were older than those without infection (71.1 vs. 66.4 years; *p* < 0.01; confidence intervals [CIs] −8.3, −1.2). AUR occurred in 44 patients (4.9%). Mean prostate volume was significantly greater among patients with AUR compared to those without (77.1 vs. 52.4 ml; *p* < 0.01; CI −40.6, −8.8). A prostate volume threshold of 79 ml was associated with increased risk of AUR.

**Conclusions:**

Perioperative antibiotics did not reduce infection rates following TPPB. Larger prostate volume is associated with an increased risk of AUR after TPPB.

## 1 INTRODUCTION

Transperineal prostate biopsy (TPPB) is a method used to sample prostate tissue to detect possible prostate cancer (PCa) in men. Traditional methods to sample the prostate were performed transrectally, where needle cores directly pierced through the rectum to obtain prostatic tissue. Although still employed in practice today, high infection and sepsis rates post‐biopsy are a critique of this method.^1^ Additionally, some evidence exists that TPPB improves detection of clinically significant PCa compared to transrectal biopsy.^2^ This is not without tradeoffs, as the rate of acute urinary retention (AUR) is greater with TPPB compared to transrectal prostate biopsy.^3^ Nevertheless, evidence has shown recent increases in the adoption of TPPB in urologic practice, largely felt to be secondary to the lower infection/sepsis rates.^4^

A multitude of studies have attempted to assess rates of infection/sepsis as well as AUR after TPPB. Current data suggest that infection rates range from 1% to 2% with sepsis rates ranging from 0.1% to 0.2%.^5^, ^6^, ^7^, ^8^ AUR is less studied, but literature estimates range 4–17% after TPPB.^9^, ^10^, ^11^, ^12^, ^13^, ^14^ Yet an elusive question remains, namely, whether perioperative antibiotic prophylaxis is required during TPPB and secondarily, what risk factors exist for the development of AUR after TPPB.

We hypothesized that perioperative antibiotic prophylaxis would not reduce infectious complications following TPPB and that larger prostate volume would be associated with increased risk of AUR. The co‐primary purpose of this study was to compare rates of infection (urinary tract infection [UTI]) after TPPB based on perioperative antibiotic prophylaxis and to assess the rate of AUR after TPPB and identify possible risk factors, with a focus on prostate size.

## 2 MATERIALS/METHODS

This was a multi‐institutional (two institutions) retrospective study with patient data spanning 2012–2025. Approval for this study was obtained from the Wake Forest University School of Medicine Institutional Review Board (IRB00133223). All patients included underwent a TPPB for elevated prostate specific antigen (PSA) with suspicion of PCa or as a confirmatory biopsy on active surveillance. Patient age at the time of biopsy was collected along with PSA, PSA density, prostate volume as determined by MRI and whether a targeted lesion was present at the time of biopsy. Use of perioperative antibiotic prophylaxis at the time of TPPB was at surgeon discretion, but when employed was a second or third generation cephalosporin (cefazolin or ceftriaxone) except in instances of a documented allergy to penicillin, in which case a different agent was employed. Dosing was not standardized, but most often 2 g, and all antibiotics were administered during induction of anaesthesia. All TPPB were performed in the operating room, with the use of monitored anaesthesia care, laryngeal mask airway or general anaesthesia, which was at the anaesthesia team's discretion.

Transrectal ultrasound guidance was employed in all instances, but TPPB templates were not standardized in the study and were at the discretion of the surgeon performing the procedure. Frequently, templates were 12 cores sampling the base, middle and apex of the prostate with two to four targeted biopsies of any lesion(s) of interest. Post‐TPPB infection was defined as least one of the following: fever (≥38.3°C) with/without symptoms of UTI or positive urine culture (>10^5^ colony forming units) within 96‐h post‐biopsy. Sepsis was defined according to previously employed methodologies as culture‐proven infection (blood or urine) plus systemic inflammatory response syndrome (temperature >38°C or <36°C; PaCO_2_ < 32 mmHg or respiratory rate >20 breaths/min; heart rate >90 beats/min; white blood cell counts >12 000/ml or <4000/ml or >10% detection of young neutrophils).^9^, ^15^ AUR was defined as an inability to urinate within 72 h of TPPB requiring catheter placement. When catheters were required for AUR, they most often stayed for 72 h, after which a voiding trial was performed. All patients were discharged with an alpha‐blocker after TPPB, irrespective of whether they were on the medication before biopsy. Specific duration of alpha‐blocker use was at provider discretion and not standardized in the study. Most patients received a phone call or healthcare chart message within the first week after TPPB to assess recovery postoperatively and screen for any potential concerning infectious symptoms. Prostate volume was determined by a board‐certified radiologist using the ellipsoid formula (length * width * height * 0.52) by preoperative magnetic resonance imaging (MRI). In cases where no MRI existed, transrectal ultrasound measurements by the surgeon performing the TPPB were employed using the ellipsoid formula. To account for patients missing MRI data who had AUR, they were excluded from any statistical test on prostate volume.

Patients were compared based on whether they received perioperative antibiotic prophylaxis using independent samples *t*‐test and chi‐squared test. Similar comparisons were conducted based on whether patients developed a UTI post‐TPPB. Observed power and beta were calculated for post‐biopsy UTI based on perioperative antibiotic administration. Lastly, patients were compared based on the development of AUR post‐TPPB using independent samples *t*‐test and chi‐squared test. A forward binary logistic regression was performed set to *p* < 0.05 with both post‐TPPB UTI and AUR as the outcomes, ensuring no collinearity between variables to assess for different variable effects on UTI and AUR. Model variables were selected based on *p* < 0.1 and/or clinical relevance. Additionally, a receiver operating characteristic curve was developed to assess the overall diagnostic performance of prostate volume on estimating the risk of AUR post‐TPPB. The Youden Index was utilized to identify a critical prostatic volume for the risk of AUR. Sensitivity, specificity, positive predictive value and negative predictive value for prostate volume on the risk of AUR were calculated.

## 3 RESULTS

Nine‐hundred‐four patients were included in the study (Table 1). The mean age at TPPB was 66.5 (±7.7) years old. The mean prostate specific antigen (PSA) at the time of TPPB in the study was 6.5 (±19) nanograms (ng)/ml and mean PSA density was 0.12 (±0.2) ng/ml/cm (cm)^3^. Mean prostate volume on MRI was 53.4 (±32.6) ml. There were 436 (48.2%) patients with a targeted lesion seen on preoperative MRI before their biopsy. The median number of TPPB cores taken was 16 (interquartile range 15–19). The rate of UTI after TPPB was 2.0% (*N* = 18), and the rate of sepsis was 0.2% (*N* = 2). The rate of AUR after TPPB was 4.9% (*N* = 44).

**TABLE 1:** The following table displays patients based on perioperative antibiotic prophylaxis.

Variable	Antibiotics	No antibiotics	*p* value	Lower	Upper
*N*	771	133			
Age (years)	66.7 (7.8)	65.3 (7.0)	0.06	−2.8	0.03
PSA (ng/ml)	3.6 (5.6)	13.5 (32.3)	0.001	4.4	15.5
PSA density (ng/ml/cm ^3^ )	0.07 (0.1)	0.2 (0.3)	<0.001	0.1	0.2
Prostate volume (ml)	53.5 (33)	52.9 (30.1)	0.8	−6.9	5.6
AUR	42 (5.4)	2 (1.5)	0.049		
Clinically significant cancer	329 (42.7)	27 (20.3)	<0.001		
UTI	15 (1.9)	3 (2.2)	0.8		
Sepsis	2 (0.3)	0	0.56		

*Note*: Relevant variables are shown in the left‐most column. Categorical variables are total numbers with percentage of the cohort in the parentheses. Continuous variables are means with standard deviations in parentheses. *p* values with confidence intervals are provided where applicable.

A total of 771 patients received perioperative antibiotics, and 133 received no perioperative antibiotics (Table 1). The mean age of patients who received antibiotics was 66.7 (±7.8) years old and 65.3 (±7.0) years old for patients that did not receive antibiotics (*p* > 0.05). Mean PSA was significantly lower in patients that received antibiotics 3.6 ng/ml (±5.6) than those who did not receive antibiotics 13.5 ng/ml (±32.3; *p* = 0.001). Mean PSA density was also significantly lower in patients who received perioperative antibiotics 0.07 (±0.1) ng/dl compared to those without antibiotics 0.2 (±0.3; *p* < 0.001). Mean prostate volume was 53.5 (±33.0) ml in those receiving antibiotics and 52.9 (±30.1) ml in those with no antibiotics (*p* > 0.05). There were 625 (81%) patients who received ceftriaxone, 104 (13.4%) patients who received cefazolin and 42 (6.6%) patients who received an alternative antibiotic regimen, which was most often ciprofloxacin. Rate of UTI was 2.3% (*N* = 3) in those without perioperative antibiotics and 1.9% (*N* = 15) in those who received perioperative antibiotics (*p* > 0.05). The rate of post‐biopsy sepsis was 0% in those without perioperative antibiotics and 0.3% (*N* = 2) in those with perioperative antibiotic prophylaxis (*p* > 0.05). Three‐hundred and twenty‐nine (41.6%) patients that received perioperative antibiotics had clinically significant cancer on final pathology, and 64 (48.1%) patients without antibiotic prophylaxis had clinically significant cancer on final pathology (*p* < 0.001). There were 42 (5.4%) cases of AUR in the antibiotic cohort and 2 (1.5%) cases of AUR in the no antibiotic cohort (*p* = 0.049). When controlling for prostate volume and AUR, older age remained a significant predictor of post‐TPPB UTI (Table S1; *p* = 0.02, odds ratio [OR] 1.1). When performing a power calculation for detection of UTI post‐TPPB based on antibiotic administration, the observed power was 0.06, and beta was 0.94, and the available sample size would be sufficient to detect an absolute risk reduction of ~2%.

As noted, 18 total patients developed a UTI with or without associated sepsis after TPPB (Table 2). The mean age of patients who developed a UTI was 71.1 (±6.2) years old and 66.4 (±7.7) years old for patients that did not receive antibiotics (*p* = 0.009). Mean PSA was equivalent in patients that developed a UTI 3.6 ng/ml (±2.8) and those who did not receive antibiotics 6.6 ng/ml (±19.0; *p* > 0.05). Mean PSA density was also equivalent in patients who developed a UTI 0.1 (±0.05) ng/ml/cm^3^ compared to those without a UTI 0.1 ng/ml/cm^3^ (±0.2; *p* > 0.05). Mean prostate volume was 67.7 (±43.8) ml in those with a UTI and 53.1 (±32.3) ml in those with no UTI (*p* > 0.05). There were 41 (4.6%) cases of AUR in the cohort with no UTI and 3 (16.7%) cases of AUR in the UTI cohort (*p* = 0.020). The mean number of cores taken during TPPB in those with a UTI was 16.8 (±3.1) and the mean number of cores taken in those without a UTI was 17.3 (4.7), which was not significantly different (*p* > 0.05).

**TABLE 2:** The following table displays patients based on development of a UTI after prostate biopsy.

Variable	UTI	No UTI	*p* value	Lower	Upper
*N*	18	886			
Age (years)	71.1 (6.2)	66.4 (7.7)	0.009	−8.3	−1.2
PSA (ng/ml)	3.6 (2.8)	6.6 (19.0)	0.6	−8.9	14.8
PSA density (ng/ml/cm ^3^ )	0.05 (0.05)	0.1 (0.2)	0.287	−0.06	0.2
Prostate volume (ml)	67.7 (43.8)	53.1 (32.3)	0.2	−37.2	8
Number biopsy cores	16.8 (3.1)	17.3 (4.7)	0.6	−1.7	2.8
AUR	3 (16.7)	41 (4.6)	0.02		
Clinically significant cancer	9 (50.0)	461 (52.0)	0.5		
Antibiotics	15 (83.3)	756 (85.3)	0.8		

*Note*: Relevant variables are shown in the left‐most column. Categorical variables are total numbers with percentage of the cohort in the parentheses. Continuous variables are means with standard deviations in parentheses. *p* values with confidence intervals are provided where applicable.

**FIGURE 1:**
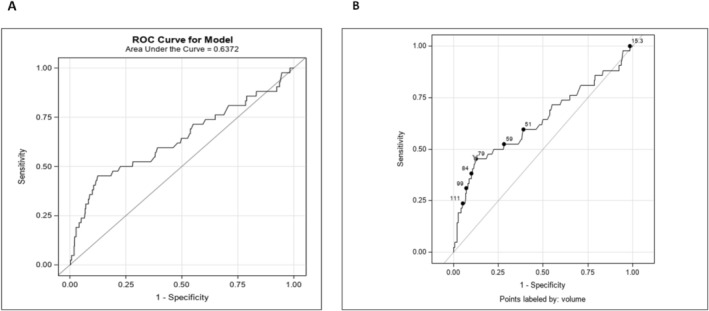
Receiver operating characteristic curve and Youden index. (A) The following figure displays receiver operating characteristic curve for prostate size on predicting risk of acute urinary retention after transperineal prostate biopsy. (B) The following figure is the Youden Index for predicting acute urinary retention after transperineal prostate biopsy. A red ‘Y’ marks the critical prostate volume where risk of acute urinary retention risk significantly increases.

Forty‐four total (4.9%) cases of AUR after TPPB were identified (Table 3). Mean patient age was 68.0 (±7.0) years old in those with AUR and 66.4 (±7.7) years old in those without AUR (*p* > 0.05). Mean PSA at the time of TPPB was 4.1 (±5.2) ng/ml and 6.6. (±19.1) ng/ml in those with and without AUR, respectively (*p* > 0.05). Likewise, mean PSA density was 0.06 (±0.1) ng/dl and 0.1 (±0.2) ng/dl in those with and without AUR, respectively (*p* > 0.05). Mean prostate volume was significantly greater in patients with AUR 77.1 (±50.5) ml compared to 52.3 (±31.0) ml (*p* = 0.003). There were 18 (2%) patients excluded from this analysis because they only had TRUS data on prostate size not MRI data. The mean number of cores taken during TPPB in those with AUR was 17 (±4.7) and in those without AUR post‐TPPB was 17.4 (4.7), which did not significantly differ (*p* > 0.05). When using prostate volume to assess risk of AUR, receiver operating curves identified the area under the curve at 0.63 (Figure 1a). The Youden Index identified a prostate volume of 79 ml as a cutoff point for increased risk of AUR (Figure 1b). The sensitivity, specificity, positive predictive value and negative predictive value of a 79 ml prostate volume for AUR were 42.9%, 95% CI (27.9, 57.8), 88% CI (85.8, 90.3), 15.8% CI (9.1, 22.5) and 96.7% CI (95.4, 98.0). When controlling for post‐TPPB UTI, prostate volume remained statistically significant between those with and without AUR after TPPB (Table S2; *p* < 0.001; OR 1.1).

**TABLE 3:** The following table displays patients based on development of acute urinary retention after prostate biopsy.

Variable	AUR	No AUR	*p* value	Lower	Upper
*N*	44	860			
Age (years)	68.0 (7.0)	66.4 (7.7)	0.178	−3.9	0.7
PSA (ng/ml)	4.1 (5.2)	6.6 (19.1)	0.584	−6.6	11.6
PSA density (ng/ml/cm ^3^ )	0.06 (0.08)	0.1 (0.2)	0.216	−0.03	0.2
Prostate volume (ml)	77.1 (50.5)	52.4 (30.9)	0.003	−40.6	−8.8
Number of biopsy cores	17.0 (4.7)	17.4 (4.7)	0.6	−1.0	1.8
Clinically significant cancer	17 (38.6)	376/812 (46.3)	0.385		
UTI	3 (6.8)	15 (1.7)	0.02		

*Note*: Relevant variables are shown in the left‐most column. Categorical variables are total numbers with percentage of the cohort in the parentheses. Continuous variables are means with standard deviations in parentheses. *p* values with confidence intervals are provided where applicable.

## 4 DISCUSSION

In this multi‐institutional review of TPPB, we identified a low risk of UTI/infection post‐biopsy and a nearly negligible risk of sepsis. Our infection rate was 2% and is within the range of prior publications.^6^, ^7^, ^16^ The rate of AUR was likewise in the range of current literature.^10^, ^17^, ^18^ Notably, lack of perioperative antibiotics did not have any association with infection after TPPB and prostate sizes were significantly larger in patients that developed AUR. In our opinion, these are the key findings in this study, with clinical relevance. This is especially true given that the European Association of Urology still includes antibiotic prophylaxis in their guidelines on prostate biopsy.^19^

Antibiotic requirements for TPPB are controversial. Prospective study has directly compared TPPB patients based on use of perioperative antibiotics. Jacewicz et al.^20^ created a randomized controlled trial (RCT) for patients undergoing TPPB comparing perioperative antibiotic prophylaxis with cefuroxime to no antibiotics. The results of that study showed no difference in UTIs or sepsis/infection requiring hospitalization between the groups. Another RCT by Chernysheva et al.^21^ found no difference in infectious complication rates based on use of perioperative antibiotics for TPPB. Castellani et al.^16^ performed a metanalysis of TPPB with and without antibiotics, focusing on infection rates. No significant difference in the rate of UTI was identified between patients based on use of antibiotics in the study, and only two cases of sepsis out of 3662 biopsies were reported.^16^ Similarly, Wolff et al.^7^ conducted a metanalysis of TPPB directly comparing antibiotics to no antibiotic prophylaxis. Over 12 000 patients were included with infection rates of 0.37% and 0.5% for those who received and did not receive antibiotics, respectively, with no significant difference identified.^7^ The infectious data presented in this manuscript add to a relatively small, but growing body of literature showing that infections after TPPB are low and perioperative antibiotic prophylaxis is not required and is relevant given an increased emphasis on antibiotic stewardship in medicine.

The rate of AUR after TPPB was not negligible, and notably prostate size was significantly greater in patients who developed AUR. Previous rates of AUR range from 4% to 17%.^13^, ^14^, ^22^ The finding of greater prostatic size in patients with AUR is in line with previous publications as well.^9^, ^10^, ^14^, ^22^ Agrawal et al.^22^ found that a preoperative prostate biopsy of 57.5 ml was the ideal cutoff for AUR, with sensitivity, specificity, positive predictive value and negative predictive value of 78.6%, 75%, 33.3% and 95.8%, respectively. In our analysis, prostate volume was good at ruling out AUR risk given the fairly high specificity and negative predictive value but poor to confirm AUR due to the low sensitivity and positive predictive value. There was also a paradoxical finding of greater rates of AUR in those who developed a UTI and a significant finding of greater AUR rates in those with antibiotics. Although this finding requires further elucidation to fully explain, it is possible that patients with more comorbidities were more likely to receive antibiotics and predisposed to develop AUR.

This study has several limitations worth acknowledging. First, it is retrospective in nature, and subject to natural biases associated with this study design, like selection bias. This is evidenced by certain baseline patient differences appreciated in the study like patient age and pre‐biopsy PSA. Furthermore, the definition of infection and sepsis used in this study is not universally accepted, and we recognize others may disagree with how these terms were defined. Our study was limited by a low overall infection rate and an unequal distribution of patients between groups, resulting in limited statistical power to detect small differences in infection risk. With observed infection rates of 2.3% in patients not receiving perioperative antibiotics and 1.9% in those receiving antibiotics, post‐hoc power to detect this 0.4% absolute difference was approximately 6%. Power analyses indicate that the available sample size would have been sufficient to detect only relatively large absolute risk reductions (~2%), but not smaller differences that may still be clinically relevant. Therefore, the absence of a statistically significant difference should be interpreted cautiously and does not exclude a modest protective or harmful effect of perioperative antibiotics. Furthermore, this does not mean the results do not hold clinical value for providers, particularly as societal guidelines continue to call for antibiotic stewardship.^23^ Fourth, although no obvious temporal differences in antibiotic administration were identified, TPPB cases were included from over a decade's span, and there are likely temporal changes in practice not fully identified in our analysis that could bias the results. The study is also subject to ascertainment bias, as those with symptoms were most likely to seek care post‐TPPB. Although the clinic call/chart message most patients received minimizes this, no standardization protocol was in place. There are also additional data points not captured that may have predisposed patients to infection or AUR, which would have strengthened analysis. This includes variables like previous alpha‐blocker usage, prior episodes of urinary retention and procedure duration. There were patients without MRI data on prostate size, and for these patients, TRUS volume was utilized, which could lead to measurement bias. However, this only applied to 2% of the study population, and we excluded these patients from analysis on prostate size and AUR. Although we recognize the results of this large, but retrospective review is not a basis alone to change clinical practice, we believe the results still hold significant value to urologists given the paucity of prospective data on this topic and need for additional data points on this important topic.

## 5 CONCLUSION

The infection rate after TPPB is low, with sepsis nearly negligible. Perioperative antibiotics were not associated with reduced infection rates in this retrospective cohort, supporting the need for prospective RCTs to definitively establish the role of antibiotic prophylaxis in TPPB. AUR is a concern with men undergoing TPPB, and those with prostate sizes of 79 ml or greater are at higher risk.

## AUTHOR CONTRIBUTIONS

*Conceptualization:* Maxwell Louis Sandberg, Robert Smith, Randall Bissette, Madeline Sandberg, Alejandro Rodriguez and Matvey Tsivian. *Data curation:* Maxwell Louis Sandberg, Robert Smith, Randall Bissette and Gregory Russell. *Formal analysis:* Maxwell Louis Sandberg, Madeline Sandberg, Gregory Russell and Matvey Tsivian. *Investigation:* Maxwell Louis Sandberg, Robert Smith, Randall Bissette, Madeline Sandberg, Gregory Russell, Alejandro Rodriguez and Matvey Tsivian. *Project administration:* Maxwell Louis Sandberg, Alejandro Rodriguez and Matvey Tsivian. *Resources:* Maxwell Louis Sandberg, Robert Smith and Matvey Tsivian. *Software:* Maxwell Louis Sandberg, Randall Bissette, Madeline Sandberg and Gregory Russell. *Supervision:* Maxwell Louis Sandberg, Alejandro Rodriguez and Matvey Tsivian. *Validation:* Maxwell Louis Sandberg and Gregory Russell. *Visualization:* Maxwell Louis Sandberg, Robert Smith, Randall Bissette, Madeline Sandberg, Gregory Russell, Alejandro Rodriguez and Matvey Tsivian. *Writing – original draft:* Maxwell Louis Sandberg. *Writing – review and editing:* Maxwell Louis Sandberg, Robert Smith, Randall Bissette, Madeline Sandberg, Gregory Russell, Alejandro Rodriguez and Matvey Tsivian. All authors critically reviewed, edited and contributed to the manuscript as well as approved the final version of the manuscript.

## CONFLICT OF INTEREST STATEMENT

The authors declare no conflicts of interest.

## Supporting information


**Table S1.** Regression model for UTI. The following table displays a forward regression model with UTI after TPPB as the outcome. Both unadjusted and adjusted odds ratios (OR) are provided. For prostate volume, effects displayed are per 1 ml increase in prostate size. S.E. represents standard error. Variables not shown in the model can be assumed to either exhibit collinearity or non‐significance.
**Table S2.** Regression model for AUR. The following table displays a forward regression model with AUR after TPPB as the outcome. Both unadjusted and adjusted odds ratios (OR) are provided. For prostate volume, effects displayed are per 1 ml increase in prostate size. S.E. represents standard error. Variables not shown in the model can be assumed to either exhibit collinearity or non‐significance.

## Data Availability

The data presented in this manuscript are not publicly available due to patient privacy concerns but are available in a limited, de‐identified format upon reasonable request to the corresponding author.
