# Supplementary material for: Perioperative antibiotic prophylaxis, prostate size and transperineal prostate biopsy
Source: BJUI Compass. 2026 May 15;7(5):e70214. doi: 10.1002/bco2.70214 (PMC13176779; doi:10.1002/bco2.70214)
Supplement: Supplementary file 1 — Table S1. Regression model for UTI. The following table displays a forward regression model with UTI after TPPB as the outcome. Both unadjusted and adjusted odds ratios (OR) are provided. For prostate volume, effects displayed are per 1 ml increase in prostate size. S.E. represents standard error. Variables not shown in the model can be assumed to either exhibit collinearity or non‐significance. Table S2. Regression model for AUR. The following table displays a forward regression model with AUR after TPPB as the outcome. Both unadjusted and adjusted odds ratios (OR) are provided. For prostate volume, effects displayed are per 1 ml increase in prostate size. S.E. represents standard error. Variables not shown in the model can be assumed to either exhibit collinearity or non‐significance. [file BCO2-7-e70214-s001.docx]

**Supplementary table 1. Regression model for UTI.** The following table displays a forward regression model with UTI after TPPB as the outcome. Both unadjusted and adjusted odds ratios (OR) are provided. For prostate volume, effects displayed are per 1 mL increase in prostate size. S.E. represents standard error. Variables not shown in the model can be assumed to either exhibit collinearity or non-significance.

| **Variable** | **Unadjusted OR** | **S.E.** | **Adjusted OR** | **Significance** | **Lower** | **Upper** |
| --- | --- | --- | --- | --- | --- | --- |
| Prostate volume (mL) | 0.006 | 0.005 | 1.0 | 0.272 | 1.0 | 1.1 |
| Age (years) | 0.09 | 0.04 | 1.1 | 0.02 | 1.1 | 1.2 |
| AUR | 1.2 | 0.7 | 3.3 | 0.082 | 0.9 | 12.6 |

**Supplementary table 2. Regression model for AUR.** The following table displays a forward regression model with AUR after TPPB as the outcome. Both unadjusted and adjusted odds ratios (OR) are provided. For prostate volume, effects displayed are per 1 mL increase in prostate size. S.E. represents standard error. Variables not shown in the model can be assumed to either exhibit collinearity or non-significance.

| **Variable** | **Unadjusted OR** | **S.E.** | **Adjusted OR** | **Significance** | **Lower** | **Upper** |
| --- | --- | --- | --- | --- | --- | --- |
| Prostate volume (mL) | 0.014 | 0.003 | 1.1 | <0.001 | 1.1 | 1.2 |
| Post-TPPB UTI | 1.3 | 0.7 | 3.6 | 0.061 | 0.9 | 13.5 |
